# Trends in Multiple Chronic Conditions Among US Adults, By Life Stage, Behavioral Risk Factor Surveillance System, 2013–2023

**DOI:** 10.5888/pcd22.240539

**Published:** 2025-04-17

**Authors:** Kathleen B. Watson, Jennifer L. Wiltz, Kunthea Nhim, Rachel B. Kaufmann, Craig W. Thomas, Kurt J. Greenlund

**Affiliations:** 1 Division of Population Health, National Center for Chronic Disease Prevention and Health Promotion, Centers for Disease Control and Prevention, Atlanta, Georgia; 2 Now with Division of Nutrition, Physical Activity, and Obesity, National Center for Chronic Disease Prevention and Health Promotion, Centers for Disease Control and Prevention, Atlanta, Georgia; 3 National Center for Chronic Disease Prevention and Health Promotion, Centers for Disease Control and Prevention, Atlanta, Georgia; 4 Department of Pediatrics, Uniformed Services University of the Health Sciences, Bethesda, Maryland; 5 Now with Office of the Assistant Secretary for Planning and Evaluation, US Department of Health and Human Services, Washington, DC; 6 Office of the Associate Director for Science, National Center for HIV, Viral Hepatitis, STD, and Tuberculosis Prevention, Centers for Disease Control and Prevention, Atlanta, Georgia

## Abstract

**Introduction:**

Chronic conditions are costly and major causes of death and disability. Addressing conditions earlier in adulthood can slow disease progression and improve well-being across the lifespan. We estimated, by life stage, 10-year trends among US adults in the prevalence of 1 or more chronic conditions, multiple chronic conditions (MCC; ≥2 conditions), and 12 selected chronic conditions.

**Methods:**

We analyzed data from the 2013–2023 (odd years) Behavioral Risk Factor Surveillance System (N = 2,673,529). We estimated the prevalence of 1 or more conditions, MCC, and each of 12 conditions, by life stage: young (18–34 y), midlife (35–64 y), and older (≥65 y) adults. We used polynomial contrasts to analyze 10-year trends.

**Results:**

In 2023, 76.4% (representing 194 million) of US adults reported 1 or more chronic conditions, including 59.5%, 78.4%, and 93.0% of young, midlife, and older adults, respectively. Moreover, 51.4% (representing 130 million) of US adults reported MCC, including 27.1%, 52.7%, and 78.8% of young, midlife, and older adults, respectively. Among young adults, from 2013 to 2023, prevalence increased significantly from 52.5% to 59.5% for 1 or more conditions and from 21.8% to 27.1% for MCC.

**Conclusion:**

Approximately 6 in 10 young, 8 in 10 midlife, and 9 in 10 older US adults report 1 or more chronic conditions. Trends in conditions worsened among young adults during 2013–2023. Recognizing the burden of chronic disease throughout life stages, especially earlier in life, practitioners and partners may consider prevention and management approaches critical for addressing costs, care, and health outcomes. Practitioners may also consider tailoring these approaches to unique roles, transitions, and challenges in different life stages.

**Summary**

**What is already known about this topic?**

Chronic conditions are costly and major causes of death and disability. Addressing conditions earlier in adulthood can slow disease progression and improve well-being across the lifespan.

**What is added by this report?**

In 2023, approximately 194 million American adults, and 6 in 10 young adults, 8 in 10 midlife adults, and 9 in 10 older adults reported 1 or more conditions. Prevalence of chronic conditions increased by 7.0 percentage points among young adults from 2013 to 2023.

**What are the implications for public practice?**

The increasing prevalence of chronic conditions earlier in adulthood can affect medical costs and quality of life. Practitioners may tailor preventive approaches to the unique roles, transitions, and challenges of different life stages.

## Introduction

Chronic conditions such as heart disease, cancer, stroke, and diabetes are costly and major causes of death and disability in the US (1,2). Over the past 20 years, the prevalence of chronic conditions has increased steadily, and this trend is expected to continue (3–5). In 2018, more than half of US adults had at least 1 chronic condition, and more than one-quarter had 2 or more chronic conditions (6). In 2016, the total direct health care costs in the US for the treatment of chronic health conditions was $1.1 trillion (7). In addition to financial costs, increased death, and disability, the burden of chronic conditions includes decreased quality of life (8), increased health care utilization (8,9), lost productivity in the workforce (10), and loss in functioning (eg, social and leisure activities) (11).

Many chronic conditions are influenced by modifiable lifestyle risk factors — excessive alcohol use, tobacco use, physical inactivity, and poor nutrition (10). Chronic conditions typically begin slowly and develop gradually over time (12). Some chronic conditions, such as high blood pressure, high cholesterol, and obesity, can lead to more serious conditions such as cardiovascular disease, chronic respiratory diseases, diabetes, some cancers, and arthritis (13).

Although age is a well-known nonmodifiable risk factor for the development of chronic conditions (14), these conditions have become more prevalent in young adults (15). With this rise in prevalence, as well as a growing aging population (1), it is increasingly important to prevent and address chronic conditions and their risk factors earlier in adulthood to help slow disease progression and improve well-being across the lifespan. Information about trends in chronic conditions, particularly across the adult life course, may help inform decision-making and programmatic efforts for effective public health actions related to the prevention and management of chronic conditions among adults of all ages.

Published information about the prevalence and trends of the number and types of chronic conditions and comorbidity by stages of life among adults (ie, young, midlife, and older adults) is outdated (5,16). Therefore, this study aimed to estimate 10-year trends (2013 to 2023) among US adults in 3 age groups in the prevalence of 1) each of 12 selected chronic conditions (asthma, arthritis, cancer, chronic kidney disease [CKD], chronic obstructive pulmonary disease [COPD], depression, diabetes, heart disease, high blood pressure, high cholesterol, obesity, and stroke), 2) 1 or more chronic conditions, and 3) multiple chronic conditions (MCC; ≥2 chronic conditions).

## Methods

We used data from the Behavioral Risk Factor Surveillance System (BRFSS) for the odd years from 2013 to 2023 for all states, the District of Columbia, and participating US territories (17). Median combined landline and cellular telephone response rates across the included years ranged from 44.0% in 2021 to 49.4% in 2019 (median, 46.2%) (18). The following states did not collect enough data to be included in the public-use data set: New Jersey (2019), Florida (2021), Kentucky (2023), and Pennsylvania (2023). The unweighted sample size across all years was 2,673,529 and ranged from 418,268 in 2019 to 491,773 in 2013. The sample sizes weighted to the noninstitutionalized adult population residing in the US and participating areas was 246,024,416 million in 2013 (minimum) and 254,139,829 in 2023 (maximum). The average percentage of missing values across the variables used in this analysis for all years was 1.3%, with the highest percentage missing for high cholesterol (13.4%) followed by obesity (8.3%).

### Chronic disease definition

Classification of chronic conditions was based on the list of conditions developed by the US Department of Health and Human Services (HHS) (19) and reported in BRFSS. Some chronic conditions listed by HHS (eg, substance abuse) were not assessed in BRFSS, so they were excluded from this analysis. Having a chronic condition was defined as responding yes to having ever been told by a doctor or other health professional that the respondent had any of the following: arthritis; cancer, excluding skin cancer (cancer); COPD; depressive disorder (depression); diabetes (excluding during pregnancy only); heart attack, angina, or coronary heart disease (heart disease); high blood pressure; high cholesterol; CKD; or stroke. Additionally, current asthma and obesity (body mass index ≥30.0, calculated as weight in kilograms divided by height in meters squared from self-reported weight and height) were included as chronic conditions.

### Statistical analyses

We estimated the prevalence of 1 or more conditions, MCC, and each selected condition for each adult life stage. We calculated the number of adults, in millions, in each category as the sample size weighted to the noninstitutionalized adult population residing in the US and participating areas (population count; weighted N) multiplied by the prevalence estimate during the corresponding year. We calculated the number of additional adults with chronic conditions as the percentage-point difference multiplied by the 2023 noninstitutionalized civilian population (weighted N). We used the following common age ranges to represent the adult life stages: young (aged 18–34 y), midlife (aged 35–64 y), and older (aged ≥65 y). We used linear and quadratic polynomial contrasts to analyze trends over time. Given the range of prevalence estimates (>1% to <90%), we defined meaningful changes based on absolute and relative changes from 2013 to 2023. Absolute changes indicate the magnitude of the change without considering the size of the starting point (ie, percentage-point change), whereas relative change indicates the degree of change relative to the starting point (ie, percentage change). The thresholds for percentage-point changes were the same thresholds used to set targets for some of the Healthy People 2030 objectives, given a relatively moderate effect size (Cohen *h* = 0.1) (20). We defined meaningfully significant (*P* < .05) changes as increasing (a prevalence ratio [PR] >1.15 or an absolute minimum difference threshold of 3 to 5 percentage points) or decreasing (a PR <0.85 or an absolute difference threshold between −3 and −5 percentage points) (20). For 2013 prevalence estimates, the threshold was 1) 5 percentage points in the range of 25% to 75%, 2) 4 percentage points in the ranges of 10% to <25% or >75% to 90%, and 3) 3 percentage points in the ranges of 1% to <10% or >90% to 99%. We used multiple imputation and the fully conditional specification method with 5 generated multiple data sets to account for missing values (1.4%) across years and measures. We used SAS version 9.4 (SAS Institute Inc) and SUDAAN version 11.0 (RTI International) to account for the complex sampling design. Survey data were also weighted to be representative of the US noninstitutionalized adult population. Institutional review board approval was not applicable for secondary analyses of publicly available BRFSS data. This activity was reviewed by the Centers for Disease Control and Prevention (CDC) and was conducted consistent with applicable federal law and CDC policy.

## Results

**Figure 1:**
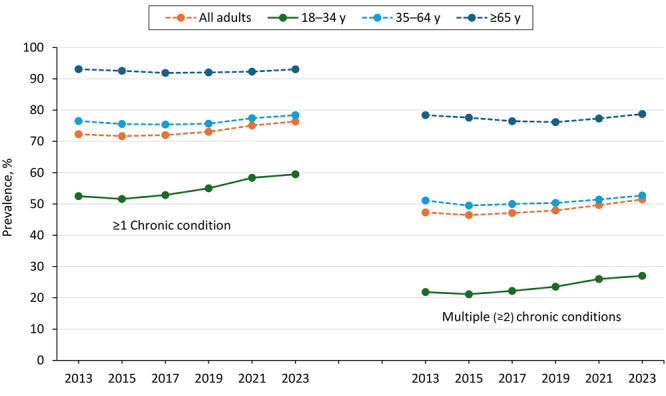
Overall and age-specific trends in the prevalence in 1 or more chronic conditions or multiple (2 or more) chronic conditions of 12 selected chronic conditions among US adults, Behavioral Risk Factor Surveillance System, 2013–2023. Linear and linear-plus-quadratic trends are significant (*P* < .05) for all groups except for adults aged ≥65 years, in which only quadratic trends are significant. The solid line indicates a meaningfully significant increasing or decreasing trend based on at least a 15% relative change or a 3- to 5-percentage–point absolute difference threshold (defined by the 2013 prevalence estimate). A dashed line indicates no meaningfully significant trend.

In 2023, 76.4% (representing more than 194 million) of US adults had at least 1 of the 12 selected chronic conditions, and 51.4% (representing 131 million) of US adults reported MCC (Table 1). By life stage, 59.5% (representing 43.7 million) of young adults, 78.4% (representing 94.8 million) of midlife adults, and 93.0% (representing 55.7 million) of older adults had 1 or more chronic conditions and one-quarter (27.1%; representing 19.9 million) of young adults, 52.7% (representing 63.8 million) of midlife adults, and 78.8% (representing 47.2 million) of older adults had MCC in 2023 (Table 2, Table 3, Table 4, and Figure 1). In 2023, the most frequently reported conditions differed across age groups. Among young adults, the most frequently reported conditions were obesity (27.3%), depression (25.0%), and high cholesterol (16.3%), whereas among midlife adults, the most frequently reported conditions were obesity (37.1%), high cholesterol (36.9%), and high blood pressure (35.0%) (Table 2 and Table 3). Older adults most frequently reported high blood pressure (61.4%), high cholesterol (55.1%), and arthritis (51.3%) (Table 4).

**Table 1:** Prevalence Estimates and 10-Year Changes in Chronic Conditions Among All US Adults, Behavioral Risk Factor Surveillance System, 2013 and 2023

Chronic condition	Prevalence in 2013 (weighted N = 246,024,416)		Prevalence in 2023 (weighted N = 254,139,829)		Change between 2023 and 2013^a^		Trend
	Has selected condition, % (95% CI)	Has selected condition and ≥1 other condition, % (95% CI)	Has selected condition, % (95% CI)	Has selected condition and ≥1 other condition, % (95% CI)	Prevalence ratio (95% CI)^b^	Percentage-point difference (95% CI)	
**Overall**							
≥1 Chronic condition	72.3 (72.0 to 72.6)	— c	76.4 (76.1 to 76.7)	— c	1.06 (1.05 to 1.06)	4.1 (3.7 to 4.4)	LQ
Multiple chronic conditions (≥2)	47.3 (47.0 to 47.6)	— c	51.4 (51.1 to 51.8)	— c	1.09 (1.08 to 1.10)	4.1 (3.7 to 4.6)	LQ
**Selected conditions**							
High cholesterol	35.2 (35.0 to 35.5)	28.9 (28.6 to 29.1)	35.3 (35.0 to 35.6)	29.9 (29.6 to 30.2)	1.00 (0.99 to 1.01)	0 (−0.4 to 0.4)	NS
High blood pressure	32.5 (32.3 to 32.8)	29.1 (28.9 to 29.4)	34.5 (34.2 to 34.8)	31.2 (30.9 to 31.5)	1.06 (1.05 to 1.07)	1.9 (1.5 to 2.4)	LQ
Obesity	28.3 (28.0 to 28.5)	21.9 (21.7 to 22.2)	32.7 (32.4 to 33.0)	25.8 (25.5 to 26.1)	1.16^d^ (1.15 to 1.17)	4.4 (4.0 to 4.8)	LQ
Arthritis	25.0 (24.8 to 25.3)	22.5 (22.3 to 22.7)	25.4 (25.1 to 25.7)	23.2 (23.0 to 23.5)	1.01 (1.00 to 1.03)	0.4 (0 to 0.7)	LQ
Depression	17.7 (17.5 to 17.9)	14.8 (14.6 to 15.0)	20.2 (19.9 to 20.4)	16.3 (16.0 to 16.5)	1.14 (1.12 to 1.15)	2.4 (2.1 to 2.8)	L
Diabetes	10.3 (10.1 to 10.5)	9.9 (9.7 to 10.1)	12.1 (11.9 to 12.3)	11.6 (11.3 to 11.8)	1.18^d^ (1.15 to 1.20)	1.8 (1.5 to 2.1)	LQ
Current asthma	9.0 (8.8 to 9.1)	7.4 (7.3 to 7.6)	9.8 (9.6 to 10.0)	8.5 (8.3 to 8.7)	1.10 (1.07 to 1.12)	0.9 (0.6 to 1.1)	LQ
Heart disease	6.7 (6.5 to 6.8)	6.5 (6.3 to 6.6)	6.5 (6.3 to 6.6)	6.3 (6.1 to 6.4)	0.97 (0.94 to 1.00)	−0.2 (−0.4 to 0)	LQ
Cancer^e^	6.5 (6.4 to 6.6)	5.8 (5.7 to 5.9)	8.0 (7.8 to 8.1)	7.2 (7.1 to 7.4)	1.22^d^ (1.20 to 1.25)	1.5 (1.3 to 1.7)	LQ
COPD	6.5 (6.3 to 6.6)	6.1 (6.0 to 6.2)	6.2 (6.1 to 6.4)	5.9 (5.8 to 6.1)	0.96 (0.93 to 0.99)	−0.3 (−0.4 to −0.1)	Q
Stroke	2.9 (2.8 to 3.0)	2.8 (2.7 to 2.9)	3.4 (3.3 to 3.5)	3.3 (3.2 to 3.4)	1.16^d^ (1.12 to 1.20)	0.5 (0.3 to 0.6)	LQ
Chronic kidney disease	2.6 (2.6 to 2.7)	2.5 (2.4 to 2.6)	3.8 (3.7 to 4.0)	3.7 (3.6 to 3.8)	1.45^d^ (1.42 to 1.48)	1.2 (1.0 to 1.3)	LQ

Abbreviations: COPD, chronic obstructive pulmonary disease; L, significant linear trend; NS, no significant linear or quadratic trends; Q, significant quadratic trend.

a First year of the trend is 2013; last year of trend is 2023.

b Prevalence ratio for the last year (2023) relative to the first year (2013) of the trend.

c Not applicable.

d Meaningfully significant changes: either a significant >15% relative change or exceeds the 3-to-5 percentage-point absolute difference threshold (defined by the 2013 prevalence estimate).

e Excludes skin cancer.

**Table 2:** Prevalence Estimates and 10-Year Changes in Chronic Conditions Among Young Adults (Aged 18 to 34 Years) in US, Behavioral Risk Factor Surveillance System, 2013 and 2023

Chronic condition	Prevalence in 2013 (weighted N = 74,492,810)		Prevalence in 2023 (weighted N = 73,385,020)		Change between 2023 and 2013^a^		Trend
	Has selected condition, % (95% CI)	Has selected condition and ≥1 other condition, % (95% CI)	Has selected condition, % (95% CI)	Has selected condition and ≥1 other condition, % (95% CI)	Prevalence ratio^b^ (95% CI)	Percentage- point difference (95%CI)	
**Overall**							
≥1 Chronic condition	52.5 (51.9 to 53.1)	— c	59.5 (58.8 to 60.1)	— c	1.13 (1.12 to 1.15)	7.0^d^ (6.1 to 7.8)	LQ
Multiple chronic conditions (≥2)	21.8 (21.4 to 22.3)	^ — c^	27.1 (26.5 to 27.6)	— c	1.24^d^ (1.21 to 1.26)	5.2^d^ (4.5 to 6.0)	LQ
**Selected conditions**							
Obesity	22.1 (21.5 to 22.6)	12.0 (11.6 to 12.4)	27.3 (26.7 to 27.8)	16.3 (15.9 to 16.8)	1.24^d^ (1.21 to 1.26)	5.2^d^ (4.5 to 6.0)^d^	L
Depression	16.4 (16.0 to 16.8)	10.7 (10.4 to 11.1)	25.0 (24.4 to 25.6)	15.9 (15.4 to 16.4)	1.53^d^ (1.50 to 1.55)	8.6^e^ (7.9 to 9.3)	LQ
High cholesterol	15.9 (15.6 to 16.3)	9.5 (9.2 to 9.8)	16.3 (15.9 to 16.8)	11.0 (10.6 to 11.4)	1.02 (0.99 to 1.06)	0.4 (−0.2 to 0.9)	Q
High blood pressure	10.4 (10.1 to 10.8)	7.9 (7.6 to 8.2)	11.5 (11.1 to 12.0)	9.3 (8.9 to 9.7)	1.10 (1.06 to 1.15)	1.1 (0.5 to 1.7)	LQ
Asthma	9.2 (8.8 to 9.5)	5.9 (5.7 to 6.2)	10.1 (9.7 to 10.5)	7.4 (7.0 to 7.7)	1.10 (1.05 to 1.15)	0.9 (0.4 to 1.5)	L
Arthritis	5.5 (5.3 to 5.8)	4.4 (4.2 to 4.6)	5.0 (4.7 to 5.3)	4.1 (3.9 to 4.4)	0.90 (0.81 to 0.98)	−0.6 (−1.0 to −0.2)	Q
COPD	2.6 (2.4 to 2.8)	2.1 (2.0 to 2.3)	1.9 (1.7 to 2.1)	1.6 (1.4 to 1.8)	0.73^d^ (0.56 to 0.89)	−0.7 (−1.0 to −0.4)	L
Diabetes	1.7 (1.6 to 1.9)	1.4 (1.3 to 1.6)	2.0 (1.7 to 2.2)	1.7 (1.5 to 2.0)	1.15^d^ (1.01 to 1.29)	0.3 (0 to 0.5)	LQ
Cancer^e^	1.4 (1.3 to 1.5)	1.1 (1.0 to 1.2)	1.0 (0.9 to 1.2)	0.8 (0.6 to 1.0)	0.74^d^ (0.47 to 1.02)	−0.4 (−0.6 to −0.1)	L
Chronic kidney disease	1.0 (0.9 to 1.2)	0.8 (0.7 to 0.9)	1.1 (0.9 to 1.3)	0.9 (0.7 to 1.0)	1.08 (0.90 to 1.26)	0.1 (−0.1 to 0.3)	NS
Heart disease	1.0 (0.9 to 1.2)	0.8 (0.7 to 1.0)	1.0 (0.9 to 1.1)	0.8 (0.7 to 1.0)	0.98 (0.78 to 1.18)	0 (−0.2 to 0.2)	Q
Stroke	0.5 (0.5 to 0.6)	0.5 (0.4 to 0.5)	0.6 (0.5 to 0.7)	0.5 (0.4 to 0.6)	1.08 (0.85 to 1.31)	0 (−0.1 to 0.2)	NS

Abbreviations: COPD, chronic obstructive pulmonary disease; L, significant linear trend; NS, no significant linear or quadratic trend; Q, significant quadratic trend.

a First year of the trend is 2013; last year of trend is 2023.

b Prevalence ratio for the last year (2023) relative to the first year (2013) of the trend.

c Not applicable.

d Meaningfully significant changes: either a significant >15% relative change or exceeds the 3-to-5 percentage-point absolute difference threshold (defined by the 2013 prevalence estimate).

e Excludes skin cancer.

**Table 3:** Prevalence Estimates and 10-Year Changes in Chronic Conditions Among Midlife Adults (Aged 35 to 64 Years) in US, Behavioral Risk Factor Surveillance System, 2013 and 2023

Chronic condition	Prevalence in 2013 (weighted N = 125,814,726)		Prevalence in 2023 (weighted N = 120,921,273)		Change between 2023 and 2013^a^		Trend
	Has selected condition, % (95% CI)	Has selected condition and ≥1 other condition, % (95% CI)	Has selected condition, % (95% CI)	Has selected condition and ≥1 other condition, % (95% CI)	Prevalence ratio (95% CI)^b^	Percentage-point difference (95% CI)	
**Overall**							
≥1 Chronic conditions	76.5 (76.2 to 76.9)	— c	78.4 (78.0 to 78.8)	— c	1.02 (1.02 to 1.03)	1.9 (1.3 to 2.4)	LQ
Multiple chronic conditions (≥2)	51.1 (50.7 to 51.5)	— c	52.7 (52.2 to 53.2)	— c	1.03 (1.02 to 1.04)	1.6 (1.0 to 2.3)	LQ
**Selected conditions**							
High cholesterol	39.3 (38.9 to 39.6)	31.9 (31.6 to 32.3)	36.9 (36.5 to 37.4)	30.6 (30.2 to 31.1)	0.94 (0.92 to 0.96)	−2.3 (−2.9 to −1.7)	LQ
High blood pressure	34.5 (34.1 to 34.9)	30.6 (30.3 to 31.0)	35.0 (34.6 to 35.5)	31.1 (30.7 to 31.6)	1.02 (1.00 to 1.03)	0.5 (0 to 1.1)	Q
Obesity	32.6 (32.2 to 33.0)	26.6 (26.2 to 26.9)	37.1 (36.7 to 37.6)	29.9 (29.5 to 30.3)	1.14 (1.12 to 1.15)	4.5 (4.0 to 5.1)	L
Arthritis	26.5 (26.2 to 26.8)	23.5 (23.2 to 23.8)	24.9 (24.5 to 25.3)	22.4 (22.0 to 22.8)	0.94 (0.92 to 0.96)	−1.6 (−2.1 to −1.1)	L
Depression	19.6 (19.4 to 19.9)	17.3 (17.0 to 17.6)	19.9 (19.6 to 20.3)	17.4 (17.1 to 17.7)	1.02 (0.99 to 1.04)	0.3 (−0.2 to 0.8)	NS
Diabetes	10.9 (10.7 to 11.2)	10.5 (10.3 to 10.8)	12.5 (12.2 to 12.9)	11.9 (11.6 to 12.2)	1.15^d^ (1.12 to 1.18)	1.6 (1.2 to 2.0)	L
Asthma	9.1 (8.9 to 9.3)	8.0 (7.8 to 8.3)	9.9 (9.7 to 10.2)	8.9 (8.7 to 9.2)	1.09 (1.06 to 1.12)	0.8 (0.5 to 1.2)	LQ
COPD	6.7 (6.5 to 6.9)	6.4 (6.2 to 6.6)	5.8 (5.6 to 6.0)	5.5 (5.3 to 5.7)	0.87 (0.81 to 0.92)	−0.9 (−1.2 to −0.6)	LQ
Heart disease	5.7 (5.6 to 5.9)	5.6 (5.4 to 5.7)	5.2 (5.0 to 5.5)	5.0 (4.8 to 5.3)	0.91 (0.85 to 0.97)	−0.5 (−0.8 to −0.2)	L
Cancer^e^	5.5 (5.3 to 5.6)	4.7 (4.6 to 4.9)	6.1 (5.9 to 6.4)	5.3 (5.1 to 5.5)	1.12 (1.08 to 1.16)	0.7 (0.4 to 0.9)	L
Stroke	2.6 (2.5 to 2.8)	2.5 (2.4 to 2.7)	3.1 (3.0 to 3.3)	3.0 (2.9 to 3.2)	1.18^d^ (1.12 to 1.24)	0.5 (0.3 to 0.7)	LQ
Chronic kidney disease	2.5 (2.4 to 2.7)	2.4 (2.3 to 2.5)	3.1 (3.0 to 3.3)	3.0 (2.9 to 3.2)	1.25^d^ (1.18 to 1.31)	0.6 (0.4 to 0.8)	L

Abbreviations: COPD, chronic obstructive pulmonary disease; L, significant linear trend; NS, no significant linear or quadratic trend; Q, significant quadratic trend.

a First year of the trend is 2013; last year of trend is 2023.

b Prevalence ratio for the last year (2023) relative to the first year (2013) of the trend.

c Not applicable.

d Meaningfully significant changes: either a significant >15% relative change or exceeds the 3-to-5 percentage-point absolute difference threshold (defined by the 2013 prevalence estimate).

e Excludes skin cancer.

**Table 4:** Prevalence Estimates and 10-Year Changes in Chronic Conditions Among Older Adults (Aged ≥65 Years) in US, Behavioral Risk Factor Surveillance System, 2013 and 2023

Chronic condition	Prevalence in 2013 (weighted N = 45,717,829)		Prevalence in 2023 (weighted N = 59,833,536)		Change between 2023 and 2013^a^		Trend
	Has selected condition, % (95% CI)	Has selected condition and ≥1 other condition, % (95% CI)	Has selected condition, % (95% CI)	Has selected condition and ≥1 other condition, % (95% CI)	Prevalence ratio (95% CI)^b^	Percentage-point difference (95% CI)	
**Overall**							
≥1 Chronic condition	93.1 (92.8 to 93.3)	— c	93.0 (92.8 to 93.3)	— c	1.00 (0.95 to 1.06)	0 (−0.4 to 0.4)	Q
Multiple chronic conditions (≥2)	78.4 (78.0 to 78.8)	— c	78.8 (78.3 to 79.3)	— c	1.00 (1.00 to 1.00)	0 (−0.4 to 0.4)	L
**Selected conditions**							
High blood pressure	63.0 (62.6 to 63.5)	59.4 (58.9 to 59.9)	61.4 (60.9 to 62.0)	58.0 (57.4 to 58.6)	0.97 (0.96 to 0.99)	−1.6 (−2.4 to −0.9)	LQ
High cholesterol	55.6 (55.1 to 56.1)	52.0 (51.5 to 52.5)	55.1 (54.6 to 55.7)	51.7 (51.1 to 52.3)	0.99 (0.98 to 1.00)	−0.5 (−1.3 to 0.3)	LQ
Arthritis	52.7 (52.2 to 53.2)	49.3 (48.8 to 49.8)	51.3 (50.8 to 51.9)	48.3 (47.8 to 48.9)	0.97 (0.96 to 0.99)	−1.4 (−2.1 to −0.6)	LQ
Obesity	26.5 (26.0 to 26.9)	25.5 (25.0 to 25.9)	30.3 (29.8 to 30.8)	29.0 (28.5 to 29.5)	1.14 (1.12 to 1.17)	3.8 (3.2 to 4.5)	L
Diabetes	22.4 (21.9 to 22.9)	21.9 (21.4 to 22.3)	23.5 (23.0 to 24.1)	22.9 (22.4 to 23.4)	1.05 (1.02 to 1.08)	1.1 (0.4 to 1.8)	L
Heart disease	18.4 (18.1 to 18.8)	18.1 (17.7 to 18.5)	15.7 (15.3 to 16.1)	15.4 (15.0 to 15.8)	0.85^d^ (0.81 to 0.89)	−2.7 (−3.3 to −2.2)	LQ
Cancer^e^	17.7 (17.3 to 18.0)	16.6 (16.2 to 17.0)	20.1 (19.7 to 20.6)	19.0 (18.6 to 19.5)	1.14 (1.11 to 1.17)	2.5 (1.9 to 3.0)	LQ
Depression	14.7 (14.3 to 15.0)	14.3 (14.0 to 14.7)	14.7 (14.3 to 15.1)	14.4 (14.0 to 14.8)	1.00 (0.97 to 1.04)	0.1 (−0.5 to 0.6)	Q
COPD	12.2 (11.8 to 12.5)	11.8 (11.5 to 12.2)	12.4 (12.0 to 12.7)	12.1 (11.7 to 12.5)	1.02 (0.98 to 1.06)	0.2 (−0.3 to 0.7)	Q
Asthma	8.3 (8.0 to 8.6)	8.1 (7.8 to 8.4)	9.3 (9.0 to 9.6)	9.0 (8.7 to 9.3)	1.12 (1.08 to 1.16)	1.0 (0.6 to 1.4)	LQ
Stroke	7.6 (7.4 to 7.9)	7.5 (7.2 to 7.7)	7.4 (7.1 to 7.7)	7.2 (7.0 to 7.5)	0.97 (0.92 to 1.03)	−0.2 (−0.6 to 0.2)	NS
Chronic kidney disease	5.7 (5.4 to 5.9)	5.6 (5.3 to 5.9)	8.6 (8.3 to 9.0)	8.5 (8.2 to 8.9)	1.52^d^ (1.48 to 1.56)	3.0 (2.5 to 3.4)	LQ

Abbreviations: COPD, chronic obstructive pulmonary disease; L, significant linear trend; NS, no significant linear or quadratic trend; Q, significant quadratic trend.

a First year of the trend is 2013; last year of trend is 2023.

b Prevalence ratio for the last year (2023) relative to the first year (2013) of the trend.

c Not applicable.

d Meaningfully significant changes: either a significant >15% relative change or exceeds the 3-to-5 percentage-point absolute difference threshold (defined by the 2013 prevalence estimate).

e Excludes skin cancer.

**Table:** 

Survey year	All adults	Adults aged 18–34 years	Adults aged 35–64 years	Adults aged 65 years or older
**≥1 Chronic conditions**				
2013	72.3	52.5	76.5	93.1
2015	71.7	51.6	75.6	92.5
2017	72.0	52.9	75.4	91.9
2019	73.0	55.0	75.6	92.0
2021	75.1	58.4	77.4	92.3
2023	76.4	59.5	78.4	93.0
**Multiple chronic conditions (≥2 conditions)**				
2013	47.3	21.8	51.1	78.4
2015	46.5	21.2	49.5	77.6
2017	47.1	22.2	50.0	76.5
2019	47.9	23.5	50.3	76.2
2021	49.6	26.0	51.4	77.3
2023	51.4	27.1	52.7	78.8

The prevalence of MCC also differed by condition and life course. For example, among adults with obesity, the likelihood of having at least 1 other condition increased with age, (Table 2, Table 3, and Table 4), from 59.7% (16.3%/27.3%) of young adults to 78.4% (29.1%/37.1%) of midlife adults and nearly all (29.0%/30.3%) older adults. Among adults who had a stroke, 8 in 10 young adults and nearly all midlife and older adults had at least 1 other condition.

### Trends in chronic conditions

During 2013–2023, the prevalence of 1 or more chronic conditions increased among young adults (52.5% to 59.5%) and remained stable (no meaningful significant trends) among midlife and older adults (Table 2, Table 3, Table 4, and Figure 1). The percentage of BRFSS respondents with MCC followed similar patterns: we found an increasing trend among young adults (21.8% to 27.1%) and stable trends for other groups. We calculated that 5.2 million more young adults had any chronic disease in 2023 than in 2013 and 3.8 million more young adults had MCC.

**Figure 2:**
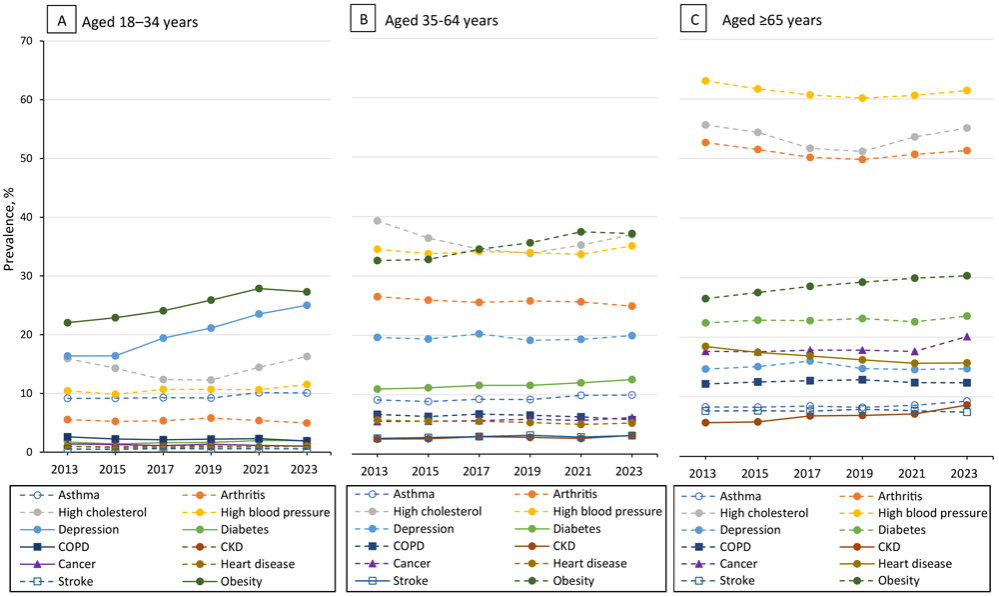
Life stage–specific trends in 12 selected chronic conditions among US adults aged 18 years or older, by age group, Behavioral Risk Factor Surveillance System, 2013–2023. Solid lines indicate meaningfully significant increasing or decreasing linear or linear-plus-quadratic trend based on at least a 15% relative change and a 3-to-5 percentage-point absolute difference threshold (defined by the 2013 prevalence estimate). Dashed lines indicate that trends are not meaningfully significant. Abbreviations: CKD, chronic kidney disease; COPD, chronic obstructive pulmonary disease.

Trends differed by condition across the adult life course (Figure 2, Table 2, Table 3, and Table 4). Among young adults, we observed meaningfully significant increasing trends for obesity (22.1% to 27.3%) and depression (16.4% to 25.0%), while trends decreased for COPD (2.6% to 1.9%) and cancer, excluding skin cancer (1.4% to 1.0%). Among midlife adults, trends increased for diabetes (10.9% to 12.5%), CKD (2.5% to 3.1%), and stroke (2.6% to 3.1%), while trends meaningfully increased for CKD (5.7% to 8.6%) and decreased for heart disease (18.4% to 15.7%) among older adults. A few conditions among young adults (high cholesterol, arthritis, heart disease), midlife adults (high blood pressure) and older adults (depression, COPD) exhibited significant quadratic trends only, thus indicating no linear change between 2013 and 2023 (Figure 2).

**Table:** 

Year	2013	2015	2017	2019	2021	2023
**Adults aged 18–34 years**						
Current asthma	9.2	9.2	9.3	9.2	10.1	10.1
Arthritis	5.5	5.2	5.3	5.8	5.4	5.0
High cholesterol	15.9	14.3	12.4	12.3	14.5	16.3
High blood pressure	10.4	9.9	10.7	10.7	10.6	11.5
Depression	16.4	16.4	19.4	21.1	23.5	25.0
Diabetes	1.7	1.4	1.6	1.7	2.0	2.0
COPD	2.6	2.2	2.1	2.2	2.3	1.9
CKD	1.0	0.9	1.1	1.0	0.9	1.1
Cancer	1.4	1.3	1.1	1.4	1.2	1.0
Heart disease	1.0	0.8	0.8	0.9	0.9	1.0
Stroke	0.5	0.5	0.6	0.6	0.6	0.6
Obesity	22.1	22.9	24.1	25.9	27.9	27.3
**Adults aged 35–64 years**						
Current asthma	9.1	8.9	9.2	9.2	9.9	9.9
Arthritis	26.5	25.9	25.6	25.8	25.6	24.9
High cholesterol	39.3	36.4	34.5	33.8	35.2	36.9
High blood pressure	34.5	33.7	34.1	33.9	33.6	35.0
Depression	19.6	19.4	20.2	19.2	19.3	19.9
Diabetes	10.9	11.2	11.6	11.6	12	12.5
COPD	6.7	6.3	6.7	6.5	6.3	5.8
CKD	2.5	2.6	2.9	2.8	2.6	3.1
Cancer	5.5	5.5	5.6	5.9	5.7	6.1
Heart disease	5.7	5.5	5.5	5.3	5.0	5.2
Stroke	2.6	2.8	3.0	3.2	2.9	3.1
Obesity	32.6	32.8	34.5	35.6	37.4	37.1
**Adults aged 65 years or older**						
Current asthma	8.3	8.3	8.5	8.2	8.6	9.3
Arthritis	52.7	51.5	50.2	49.8	50.7	51.3
High cholesterol	55.6	54.4	51.7	51.2	53.6	55.1
High blood pressure	63.0	61.7	60.7	60.1	60.6	61.4
Depression	14.7	15.1	16.0	14.8	14.6	14.7
Diabetes	22.4	22.9	22.8	23.1	22.6	23.5
COPD	12.2	12.5	12.7	12.9	12.4	12.4
CKD	5.7	5.8	6.8	6.9	7.1	8.6
Cancer	17.7	17.6	17.8	17.8	17.6	20.1
Heart disease	18.4	17.4	16.9	16.2	15.6	15.7
Stroke	7.6	7.7	7.6	7.9	7.7	7.4
Obesity	26.5	27.5	28.5	29.2	29.9	30.3

## Discussion

In 2023, approximately 6 in 10 young adults (59.5%), 8 in 10 midlife adults (78.4%), and 9 in 10 older adults (93.0%) reported at least 1 chronic condition. These percentages translate to 43.7 million young adults, 94.8 million midlife adults, 55.7 million older US adults, and more than 194 million US adults overall. Moreover, 1 in 4 young adults (27.1%; 19.9 million), 2 in 4 midlife adults (52.7%; 63.8 million), and 3 in 4 older adults (78.8%; 47.2 million) reported MCC. During 2013–2023, estimates for the prevalence of young adults with chronic conditions represent a meaningful increase from 52.5% to 59.5% for at least 1 condition and from 21.8% to 27.1% for MCC. This meaningful increase in 1 or more or MCC among young adults no longer remained when the 2 most common conditions (obesity and depression) were excluded, based on sensitivity analysis using data in Table 2. Although trends increased among midlife adults and increased or remained stable among older adults, these changes were very small. Regardless, these findings are cause for concern. They demonstrate the widespread lack of noticeable improvement in reducing the prevalence of 1 or more chronic conditions or MCC among adults across the life course, particularly among young adults and particularly as a result of rises in obesity and depression.

In 2018, a US national survey reported prevalence estimates for 1 or more chronic conditions and MCC among adults aged 18 to 44 years, 45 to 64 years, and 65 years or older (6). In the youngest group, 27% reported 1 or more conditions and 7% reported MCC. In the midlife group, 63% reported 1 or more conditions and 33% reported MCC, and in the oldest age group, 88% reported 1 or more conditions and 64% reported MCC. Data from 1999–2000 to 2017–2020 also showed a stable trend among US adults without hypertension; however, MCC increased among adults with hypertension (21). When limited to years (2013–2014 through 2017–2020) that overlapped with our study, trends among both groups (adults with and without hypertension) remained stable (21). Although differences exist in the magnitude of the prevalence of MCCs between our study and other studies, they can be at least partially explained by differences in the chronic conditions included between studies. For example, hepatitis was a condition included in the estimates from 2018 (6) and 1999–2000 to 2017–2020 (21); high cholesterol, depression, and obesity were included only in the estimates from 1999–2000 to 2017–2020 (21) and our study; and high blood pressure was included only in the estimates from 2018 (6) and our study. Some of these conditions (ie, obesity and high cholesterol) are more common and increasing compared with hepatitis (21).

The 3 most common conditions varied somewhat across the life course. Young adults most frequently reported obesity, depression, and high cholesterol, while midlife adults reported high blood pressure, high cholesterol, and obesity. Older adults most frequently reported high blood pressure, high cholesterol, and arthritis. Most chronic conditions at every life stage have common modifiable lifestyle factors (ie, physical inactivity, poor nutrition, tobacco use, and excessive alcohol use), for which evidence-based public health and clinical interventions are available (1,22–24). The role of medical professionals in supporting lifestyle behavior changes is an important part of prevention and treatment (1), particularly as lifestyle medicine has recently been recognized as a medical specialty (25). The overlaps in most common chronic conditions by life stage and the increasing prevalence among younger adults suggest that efforts for prevention, treatment, and management of chronic conditions may need to be strengthened, especially for younger adults. However, access to care and healthier lifestyles can vary by life stage. For example, a midlife adult may be a caregiver to children as well as aging parents (26) and thus may not have time to be involved in a healthy lifestyle. Environmental factors affect chronic disease prevention and management by limiting opportunities to make healthy lifestyle choices. For example, in 2023, compared with their counterparts, a higher proportion of adults 18 to 44 years reported living in poverty (27). Low-income individuals are more likely to live in communities where residents face difficulty accessing recreation opportunities and healthier foods (28).

Younger adults are more likely than older adults to lack health insurance consistently, a factor that makes it challenging to get preventive screenings or specialist follow-up care (25). Young adults may also face an adult health care system that may not be as responsive to their developmental stage or have concerns about confidentiality (15). Health interventions and programs that include a developmental perspective and incorporate mechanisms tailored to young adults may help improve the prevalence of chronic conditions at this life stage (15). Regardless of age, avoiding and addressing these risk behaviors, as well as following screening guidelines, are critical for reducing the prevalence of chronic conditions across the adult life course and improving the likelihood of decreased illness and early death (1,23).

Across all age groups, adults with MCC have unique challenges: some clusters of conditions may interact such that the combined health outcome of the conditions is greater than the effects of the conditions individually (29,30). For example, having depression in conjunction with another chronic condition may present overlapping symptoms or interfere with the treatment of either depression or the other chronic condition (31). Additionally, some individuals with comorbid conditions may receive care for one condition but not the other (29,32). Problems with coordination of care between primary care physicians and specialists can result in delays in diagnosis and health issues unintentionally caused by medical treatments (33). The 2010 HHS framework for MCC offers goals and strategies to address these issues and optimize the health and quality of life for people with MCC (29).

### Limitations

Although the large sample size and sample design allowed for generalizability to the noninstitutionalized adult population, the findings in this study are subject to at least 3 limitations. First, BRFSS data are self-reported and subject to recall and social desirability biases. For example, the prevalence of physician-diagnosed chronic conditions ascertained by self-report might be underestimated; however, at the state-level, the prevalence of some conditions is consistent with estimates obtained from electronic health records (34). Second, the median response rate of 46.6% might reduce generalizability; however, BRFSS uses a sophisticated weighting method (iterative proportional fitting) that does not require demographic information for small geographic areas, thereby reducing the potential for certain biases (35). Lastly, trends by sociodemographic factors and health risk behaviors were not assessed in this study.

### Public health implications

This study updates national data on the prevalence of selected chronic conditions and MCC among US adults by age group and contributes to the growing body of evidence demonstrating the increase in chronic conditions earlier in life — among young adults (aged 18–34 y) (15,36). While the lifespan in the US has been increasing, the health span (the period of life spent in good health) has lagged (37). As the cohort of young adults ages, worsening health outcomes will increase the burden of care on communities, clinicians, and health care delivery systems (15). Programs tailored to address depression, obesity, and high cholesterol among young adults may be particularly effective in reducing the burden of chronic disease as they enter middle age and older adulthood; and policies aimed at improving the upstream social determinants of health (built environment, food insecurity, and access to health care) would drive sustained improvements in the health of aging populations (10). Future work on the progression of MCC at the individual and population levels is critical to developing strategies that mitigate the potential surge in the prevalence of MCC resulting from younger populations beginning to experience age-related chronic diseases.

### Conclusion

Multiple chronic conditions affect more than half (128 million) of US adults, and more than three-quarters (191 million) of US adults have at least 1 of the 12 chronic conditions included in this study. Trends in the prevalence of 1 or more chronic conditions from 2013 to 2023 worsened among young and midlife adult populations and showed less than a 1% improvement (percentage-point difference <1) among older adults. Trends in conditions varied by condition and life stage. Coordinated efforts by public and private sectors might help improve the availability of evidence-based interventions, policies, and programs to prevent, treat, and manage chronic conditions among adults in different situations and stages of life.

## References

[1] Hacker K . The burden of chronic disease. *Mayo Clin Proc Innov Qual Outcomes.* 2024;8(1):112–119. 10.1016/j.mayocpiqo.2023.08.005 38304166 PMC10830426

[2] Kochanek KD , Murphy BS , Xu J , Arias E . Mortality in the United States. *NCHS Data Brief.* 2022;(492):1–8. 36598387

[3] Mattke S , Mengistu T , Klautzer L , Sloss EM , Brook RH . Improving care for chronic conditions: current practices and future trends in health plan programs. *Rand Health Q.* 2015;5(2):3. 28083379 10.7249/rhq-05-02-03 PMC5158283

[4] Mohebi R , Chen C , Ibrahim NE , McCarthy CP , Gaggin HK , Singer DE , . Cardiovascular disease projections in the United States based on the 2020 census estimates. *J Am Coll Cardiol.* 2022;80(6):565–578. 10.1016/j.jacc.2022.05.033 35926929 PMC9396356

[5] Omura JD , Hyde ET , Imperatore G , Loustalot F , Murphy L , Puckett M , . Trends in meeting the aerobic physical activity guideline among adults with and without select chronic health conditions, United States, 1998–2018. *J Phys Act Health.* 2021;18(S1):S53–S63. 10.1123/jpah.2021-0178 34465653 PMC10977617

[6] Boersma P , Black LI , Ward BW . Prevalence of multiple chronic conditions among US adults, 2018. *Prev Chronic Dis.* 2020;17:E106. 10.5888/pcd17.200130 32945769 PMC7553211

[7] Waters H , Graf M . The costs of chronic disease in the U.S. Milken Institute. August 28, 2018. Accessed January 25, 2025. https://milkeninstitute.org/content-hub/research-and-reports/reports/costs-chronic-disease-us

[8] Makovski TT , Schmitz S , Zeegers MP , Stranges S , van den Akker M . Multimorbidity and quality of life: systematic literature review and meta-analysis. *Ageing Res Rev.* 2019;53:100903. 10.1016/j.arr.2019.04.005 31048032

[9] Allegrante JP , Wells MT , Peterson JC . Interventions to support behavioral self-management of chronic diseases. *Annu Rev Public Health.* 2019;40(1):127–146. 10.1146/annurev-publhealth-040218-044008 30601717 PMC6684026

[10] Bauer UE , Briss PA , Goodman RA , Bowman BA . Prevention of chronic disease in the 21st century: elimination of the leading preventable causes of premature death and disability in the USA. *Lancet.* 2014;384(9937):45–52. 10.1016/S0140-6736(14)60648-6 24996589

[11] Boudewijns EA , Claessens D , van Schayck OCP , Keijsers LCEM , Salomé PL , In ’t Veen JCCM , . ABC-tool reinvented: development of a disease-specific ‘Assessment of Burden of Chronic Conditions (ABCC)-tool’ for multiple chronic conditions. *BMC Fam Pract.* 2020;21(1):11. 10.1186/s12875-019-1075-8 31931729 PMC6958572

[12] Snyderman R , Sanders Williams R . Prospective medicine: the next health care transformation. *Acad Med.* 2003;78(11):1079–1084. 10.1097/00001888-200311000-00002 14604864

[13] World Health Organization. Noncommunicable diseases. December 23, 2024. Accessed June 17, 2024. https://www.who.int/news-room/fact-sheets/detail/noncommunicable-diseases

[14] Harris RE . Global epidemiology of chronic diseases: the epidemiologic transition. In: Harris RE, ed. *Epidemiology of Chronic Disease: Global Perspectives.* 1st ed. Jones and Bartlett Learning; 2012:1–21.

[15] Committee on Improving the Health, Safety, and Well-Being of Young Adults; Board on Children, Youth, Families; Institute of Medicine, National Research Council. Young adults in the 21st century. In: Bonnie RJ, Stroud C, Breiner H, eds. *Investing in the Health and Well-Being of Young Adults.* National Academies Press; 2015:35–75. 25855847

[16] Buttorff C , Ruder T , Bauman M . *Multiple Chronic Conditions in the United States.* RAND Corporation; 2017.

[17] Centers for Disease Control and Prevention. BRFSS overview. Accessed August 10, 2024. https://www.cdc.gov/brfss/annual_data/2023/pdf/Overview_2023-508.pdf

[18] Centers for Disease Control and Prevention. BRFSS combined landline and cell phone weighted response rates by state, 2023. April 25, 2013. Accessed September 12, 2024. https://www.cdc.gov/brfss/annual_data/2023/pdf/2023_ResponseRates_Table-508.pdf

[19] Goodman RA , Posner SF , Huang ES , Parekh AK , Koh HK . Defining and measuring chronic conditions: imperatives for research, policy, program, and practice. *Prev Chronic Dis.* 2013;10:E66. 10.5888/pcd10.120239 23618546 PMC3652713

[20] Hubbard K , Talih M , Klein RJ , Huang DT . Target-setting methods in Healthy People 2030. Healthy People Statistical Notes, no 28. National Center for Health Statistics; 2020.

[21] Alanaeme CJ , Ghazi L , Akinyelure OP , Wen Y , Christenson A , Poudel B , . Trends in the prevalence of multiple chronic conditions among US adults with hypertension from 1999–2000 through 2017–2020. *Am J Hypertens.* 2024;37(7):493–502. 10.1093/ajh/hpae040 38576398 PMC11519032

[22] Jacobs JA , Jones E , Gabella BA , Spring B , Brownson RC . Tools for implementing an evidence-based approach in public health practice. *Prev Chronic Dis.* 2012;9:E116. 10.5888/pcd9.110324 22721501 PMC3457760

[23] Kris-Etherton PM , Petersen KS , Després JP , Braun L , de Ferranti SD , Furie KL , . Special considerations for healthy lifestyle promotion across the life span in clinical settings: a science advisory from the American Heart Association. *Circulation.* 2021;144(24):e515–e532. 10.1161/CIR.0000000000001014 34689570

[24] The Community Guide. The Guide to Community Preventive Services. Accessed July 17, 2024. https://www.thecommunityguide.org

[25] Rosenfeld RM . Physician attitudes on the status, value, and future of board certification in lifestyle medicine. *Am J Lifestyle Med.* 2022;18(1):118–130. 10.1177/15598276221131524 39184265 PMC11339759

[26] Infurna FJ , Staben OE , Lachman ME , Gerstorf D . Historical change in midlife health, well-being, and despair: cross-cultural and socioeconomic comparisons. *Am Psychol.* 2021;76(6):870–887. 10.1037/amp0000817 34914427 PMC9377163

[27] US Department of Health and Human Services. Economic stability — Healthy People 2030. 2020. Accessed December 10, 2024. https://odphp.health.gov/healthypeople/objectives-and-data/browse-objectives/economic-stability

[28] Lee V , Mikkelsen L , Srikantharajah J , Cohen L . *Strategies for Enhancing The Built Environment to Support Healthy Eating and Active Living.* Prevention Institute; 2008.

[29] Parekh AK , Goodman RA , Gordon C , Koh HK ; HHS Interagency Workgroup on Multiple Chronic Conditions. Managing multiple chronic conditions: a strategic framework for improving health outcomes and quality of life. *Public Health Rep.* 2011;126(4):460–471. 10.1177/003335491112600403 21800741 PMC3115206

[30] Wolff JL , Starfield B , Anderson G . Prevalence, expenditures, and complications of multiple chronic conditions in the elderly. *Arch Intern Med.* 2002;162(20):2269–2276. 10.1001/archinte.162.20.2269 12418941

[31] Gold SM , Köhler-Forsberg O , Moss-Morris R , Mehnert A , Miranda JJ , Bullinger M , . Comorbid depression in medical diseases. *Nat Rev Dis Primers.* 2020;6(1):69. 10.1038/s41572-020-0200-2 32820163

[32] Institute of Medicine (US) Committee on Quality of Health Care in America. *Crossing the Quality Chasm: A New Health System for the 21st Century.* National Academies Press; 2001. 25057539

[33] Kim B , Lucatorto MA , Hawthorne K , Hersh J , Myers R , Elwy AR , . Care coordination between specialty care and primary care: a focus group study of provider perspectives on strong practices and improvement opportunities. *J Multidiscip Healthc.* 2015;8:47–58. 10.2147/JMDH.S73469 25653538 PMC4310270

[34] Klompas M , Cocoros NM , Menchaca JT , Erani D , Hafer E , Herrick B , . State and local chronic disease surveillance using electronic health record systems. *Am J Public Health.* 2017;107(9):1406–1412. 10.2105/AJPH.2017.303874 28727539 PMC5551591

[35] Centers for Disease Control and Prevention. Methodologic changes in the Behavioral Risk Factor Surveillance System in 2011 and potential effects on prevalence estimates. *MMWR Morb Mortal Wkly Rep.* 2012;61(22):410–413. 22672976

[36] Watson KB , Carlson SA , Loustalot F , Town M , Eke PI , Thomas CW , . Chronic conditions among adults aged 18–34 years — United States, 2019. *MMWR Morb Mortal Wkly Rep.* 2022;71(30):964–970. 10.15585/mmwr.mm7130a3 35900929 PMC9345173

[37] Olshansky SJ . From lifespan to healthspan. *JAMA.* 2018;320(13):1323–1324. 10.1001/jama.2018.12621 30242384

